# Strain engineering for improved expression of recombinant proteins in bacteria

**DOI:** 10.1186/1475-2859-10-32

**Published:** 2011-05-14

**Authors:** Tomohiro Makino, Georgios Skretas, George Georgiou

**Affiliations:** 1 Department of Chemical Engineering, The University of Texas at Austin, Austin, Texas 78712, USA; 2 Institute for Cellular and Molecular Biology, The University of Texas at Austin, Austin, Texas 78712, USA; 3 Department of Biomedical Engineering, The University of Texas at Austin, Austin, Texas 78712, USA; 4 Section of Molecular Genetics and Microbiology, The University of Texas at Austin, Austin, Texas 78712, USA; 5 Insitute of Biological Research and Biotechnology, National Hellenic Research Foundation, 48 Vassileos Constantinou Ave., 11635 Athens, Greece; 6 Asubio Pharma CO., LTD. 6-4-3, Minatojima-Minamimachi Chuo-ku, Kobe 650-0047, Japan

## Abstract

Protein expression in *Escherichia coli *represents the most facile approach for the preparation of non-glycosylated proteins for analytical and preparative purposes. So far, the optimization of recombinant expression has largely remained a matter of trial and error and has relied upon varying parameters, such as expression vector, media composition, growth temperature and chaperone co-expression. Recently several new approaches for the genome-scale engineering of *E. coli *to enhance recombinant protein expression have been developed. These methodologies now enable the generation of optimized *E. coli *expression strains in a manner analogous to metabolic engineering for the synthesis of low-molecular-weight compounds. In this review, we provide an overview of strain engineering approaches useful for enhancing the expression of hard-to-produce proteins, including heterologous membrane proteins.

## Introduction

Since the beginning of the modern biotechnology era in the late 70s, *Escherichia coli *has been used extensively for protein overexpression due to its rapid growth rate, ease of high-cell-density fermentation, low cost and, most importantly, the availability of excellent genetic tools. The optimization of recombinant protein expression in *E. coli *has been carried out largely by trial and error by varying simple parameters such as expression vectors, host strains, media composition, and growth temperature.

During the past years, extensive studies have shown that the replacement of codons within a heterologous gene with synonymous ones used preferentially in the expression host (codon optimization), and the manipulation of the nucleotide sequence of the translational initiation region can have a profound effect on recombinant protein yields [1-4]. mRNA secondary structures, RNase cleavage sites and ribosome-binding site sequestering sequences have been introduced into expression constructs in efforts to increase mRNA stability, improve transcription termination and translation efficiency [5]. Currently, a wide selection of commercially available expression vectors is provided with different origins of replication, different promoters, translation initiation regions, antibiotic resistance markers, transcription terminators, etc. The selection of the proper vector together with the use of codon-optimized genes [6,7] is in many instances sufficient to enable the accumulation of the target protein at an appreciable level. This optimization strategy, however, does not address problems related to protein misfolding and solubility. Trial and error optimization of growth temperature, media optimization of induction conditions, the use of fusions to solubilizing partners and chaperone co-expression have to be deployed to achieve better yields of biologically active product. For example, fusions of the protein of interest with partners, such as the maltose-binding protein (MBP) or glutathione-S-transferase (GST) [8-10], as well as co-expression of proteins that can assist in folding, notably molecular chaperones/co-chaperones (GroEL/GroES, DnaK/DnaJ etc) [11], are used routinely to increase soluble protein yields. Nevertheless, there are many proteins for which none of these approaches are effective.

Directed evolution of the polypeptide sequence for improved synthesis and folding in a prokaryotic host, also termed as "expression maturation", has been employed successfully for a variety of complex heterologous proteins including mammalian G protein-coupled receptors (GPCR), hemoglobin, antibody fragments and other proteins [12-15]. In expression maturation, the gene encoding the target protein is subjected to random mutagenesis (e.g. by error-prone PCR), the library of mutant genes is expressed, and variants with increased solubility are identified, either by applying selective pressure or by high-throughput screening [12-15]. The limitations of this approach are first, that it lacks generality since it needs to be applied for every individual protein target; second, the need for a high-throughput screen for expression applicable to the protein of interest; and third, the concern that the selected mutations may also affect the function, stability, or the structure of the protein.

One alternative to expression maturation is to engineer host strains that are suitable for the expression of particular classes of proteins, such as proteins with complex disulfide topologies, membrane proteins, or proteins with intrinsically slow folding kinetics, which, in general, are more prone to misfolding and aggregation. The advantage of this approach is its broader generality since it leads to the generation of high-expression strains for a variety of polypeptides that share some common features. Furthermore, analysis of the chromosomal or vector mutations that confer enhanced expression can provide a better understanding of the rate limiting steps in protein expression and perhaps be of general utility for the production of other similar proteins.

Here, we will provide a review of current efforts to enhance recombinant protein production in *E. coli *through genetic and genome-scale engineering. Relevant technologies for the creation and isolation of overexpressing mutants and successful examples of increased protein yields are presented. The terms "genetic engineering" and "strain engineering" are used interchangeably throughout this text.

### Strain/genetic engineering for enhanced protein expression in bacteria

Chromosomal lesions such as nucleotide substitutions, gene deletions or insertions and, alternatively, overexpression of homologous or heterologous genes can all influence the expression of target proteins. Genetic modifications can be introduced into DNA in a targeted manner within a specific cellular pathway known to be involved in protein biogenesis. Alternatively, when the causes of poor expression are not known, a library of random chromosomal gene fragments can be cloned and co-expressed with the target protein or, the entire genome may be subjected to random mutagenesis, followed by screening to isolate clones that confer increased protein production.

### 1. Targeted strain engineering strategies

Targeted strain engineering focuses on the introduction of mutations in DNA sequences known to affect protein synthesis, degradation, secretion or folding. Several excellent reviews describing the strategies for improving protein secretion or for limiting protein degradation have already been published [16-19]. Therefore, we will focus here only on the engineering of bacterial strains for improved protein synthesis and/or folding.

#### 1.1 Engineering of mRNA stability and translational efficiency

In bacteria, the half-life of mRNA is much shorter than in eukaryotic cells and can be the rate limiting step in translation and, hence, in protein synthesis. The endonuclease RNaseE catalyzes the first, rate-determining step in the cleavage of numerous transcripts in *E. coli*. Mutations, such as the well characterized *rne131 *allele, that attenuate the activity of this essential protein, confer increased mRNA stability, which can in turn result in higher protein expression levels [20]. A BL21 derivative strain carrying the *rne131 *allele is commercially available by Invitrogen under the brand name BL21 Star™.

As mentioned briefly above, translational efficiency can be dramatically affected by codon usage and by the sequence of the translation initiation region. Numerous reports have demonstrated that the use of engineered strains that co-express tRNAs for rare codons such as the Rosetta™ strains from Invitrogen and the BL21 CodonPlus strains from Novagen can enhance recombinant protein production significantly [21,22].

#### 1.2 Improving protein folding by chaperone co-expression

A common, and occasionally successful, strategy for preventing protein aggregation is the co-expression of molecular chaperones. The biochemistry and mechanism of action of bacterial molecular chaperones and enzymes that assist folding have been reviewed previously [23], and will not be covered in detail here. It is important to note that folding factors such as DnaK/DnaJ/GrpE, GroEL/GroES, IbpA/IbpB, Skp, trigger factor and FkpA have been used successfully to prevent protein aggregation of cytoplasmic or periplasmic proteins [24-28]. The latter two proteins also display X-Pro isomerization activity but their function in assisting protein folding has been attributed primarily to their role as chaperones [29,30]. DnaK/DnaJ/GrpE, GroEL/GroES and ClpB can function synergistically in assisting protein folding and therefore expression of these chaperones in combinations has been shown to be beneficial for protein expression [11,31].

#### 1.3 Expression of disulfide-bonded proteins

Many biotechnologically important proteins contain disulfide bonds. The cytoplasm of *E. coli *is normally maintained in a reduced state that precludes the formation of disulfide bonds via the action of the thioredoxin and glutaredoxin/glutathione enzyme systems [32]. Therefore, proteins with disulfides normally need to be exported into the periplasm. In the periplasm, disulfide bond formation and isomerization is catalyzed by the Dsb system, which comprises DsbABCD and G. Co-expression of the cysteine oxidase DsbA, the disulfide isomerase DsbC or combinations of the Dsb proteins, have been employed for the successful expression of numerous heterologous proteins such as scFvs, plasminogen activators, human nerve growth factors and others [25,33-35].

Mutant strains defective in glutathione reductase (*gor*) or glutathione synthetase (*gshA*) together with thioredoxin reductase (*trxB*) render the cytoplasm oxidizing. These strains are unable to reduce ribonucleotides and therefore cannot grow in the absence of exogenous reductant, such as dithiothreitol (DTT). Suppressor mutations in the gene *ahpC*, which encodes the peroxiredoxin AhpC, allow the channeling of electrons onto the enzyme ribonulceotide reductase enabling the cells to grow in the absence of DTT. In such strains, exposed protein cysteines become readily oxidized in a process that is catalyzed by thioredoxins, in a reversal of their physiological function, resulting in the formation of disulfide bonds. A number of heterologous multi-disulfide-bonded proteins have been produced in the cytoplasm of *E. coli *FA113 cells (trxB gor ahpC*) or Origami™ (Novagen) at high yields [36]. Additionally, it was recently shown that bacterial strains with different mutations in the thioredoxin/thioredoxin reductase and glutaredoxin/glutathione reductase genes and containing different suppressor mutations in alleles of *ahpC*, display dramatic differences in the kinetics of cysteine oxidation in the cytoplasm and in the yield of correctly folded proteins [28,37].

Very recently, Ruddock and colleagues have shown that overexpression of the sulfhydryl oxidase Erv1p from the inner membrane space of yeast mitochondria enables high-level production of a variety of complex, disulfide-bonded proteins of eukaryotic origin in the cytoplasm of *E. coli *[38]. Remarkably, these investigators found that disulfide bond formation upon Erv1p co-expression could take place even in the absence of *trxB gor *mutations [39].

#### 1.4 Glycoprotein production in *E. coli*

Until recently, protein glycosylation was considered a post-translational modification which can only be carried out in eukaryotes. In 2002, it was discovered that the enteropathogenic bacterium *Campylobacter jejuni *can perform protein *N*-glycosylation. Subsequent transfer of the *pgl *locus led to the development of *E. coli *strains which could perform *N*-glycosylation of the *C. jejuni *proteins AcrA and PEB3 [40]. The *pgl *locus consists of five putative glycosyltransferases (*pglACHIJ*), an oligosaccharyl transferase (*pglB*), four enzymes involved in sugar biosynthesis (*galE, pglDEF*), and a flippase (*wlaB*) [41,42]. *pglB *mutants having relaxed specificity have been engineered [41], thus opening the way for the incorporation of diverse glycan structures onto a target polypeptide. Furthermore, forward engineering using shotgun proteomics and metabolic flux analysis has been applied to significantly improve the efficiency of protein glycosylation in *E. coli *[43]. Several groups have started to utilize the *C. jejuni *pgl *N*-linked glycosylation platform for biotechnological applications, including the generation of glyco-conjugated vaccines in bacteria [44,45]. Very recently, two groups have reported the display of glycoproteins onto filamentous phage, which in turn may enable the isolation of novel types of glycoproteins from combinatorial libraries [46,47].

#### 1.5 Acetylated protein production in *E. coli*

Acetylation is a very commonly encountered protein modification, which is important for regulation in key cellular processes [48,49]. In eukaryotes, most proteins are acetylated at the alpha-amino group of the N-terminal amino acid or at the epsilon-amino group of internal lysines. In general, eukaryotic N-terminal acetylation is carried out by specific *N*-α-acetyltransferase (Nat) complexes and is thought to take place co-translationally at the ribosome [50]. This protein modification, however, is rarely encountered in bacteria, and in contrast to eukaryotes, it takes place in a post-translational manner [51]. In a very recent study, overexpression of the bacterial *N*-α-acetyltransferase RimJ was found to be sufficient for the production of fully acetylated recombinant thymosin alpha 1 in *E. coli *[52]. Even more recently, Mulvihill and coworkers demonstrated that co-expression of one of the members of the Nat complex of the fission yeast (NatB) with its target substrate proteins could successfully produce a number of acetylated proteins of human and yeast origin in *E. coli *[53]. These findings demonstrate that a wide variety of acetylated proteins could be potentially produced recombinantly in *E. coli*.

### 2. Global genetic/strain engineering

Strains that confer improved protein expression can be engineered by screening libraries of chromosomal mutants or plasmid-encoded expression libraries of heterologous or native genes. An important advantage of this approach is that no *a priori *hypotheses or extensive knowledge regarding bottlenecks in recombinant protein expression is required. Identification and analysis of the effects of the genetic lesions isolated in this process can in turn provide a better understanding of the pathways that limit the expression of the desired protein. The key factors for successful strain engineering by library screening approaches are: 1) the type of genetic modification applied 2) the quality of the constructed library, and 3) the availability of a high-throughput screen that can correctly identify clones displaying the desired phenotype.

Libraries of bacteria containing lesions randomly distributed over the entire chromosome can be readily generated by classical mutagenesis methods, such as UV irradiation, chemical mutagens, and random transposon mutagenesis. A very useful tool for studying the effect of gene knockouts on recombinant protein expression and other properties/phenotypes is the Keio collection, a publicly available library of all single knockouts of all the non-essential *E. coli *K-12 genes [54].

In addition to the classical mutagenesis strategies, new techniques for genome engineering have been developed recently for generating libraries in which the expression of chromosomally encoded genes can been up- or down-regulated. These techniques include global transcription machinery engineering (gTME) [55] and trackable multiplex recombineering (TRMR) [56]. These and other genome engineering technologies may be employed to access phenotypes that may be difficult to obtain via classical mutagenesis approaches [57].

#### 2.1 Strain engineering by classical mutagenesis

One of the most frequently encountered phenotypic consequences of recombinant protein expression is growth retardation or complete growth arrest of the host following induction of gene overexpression. More than a decade ago, Walker and coworkers isolated *E. coli *BL21(DE3) mutant strains carrying spontaneously acquired suppressor mutations that alleviate the toxicity caused by the production of cytotoxic proteins under the control of the strong T7 promoter [58]. These strains, which are called C41 and C43 or "Walker strains", are widely used to produce increased levels of hard-to-express proteins primarily because they allow increased biomass production. Not surprisingly, it was later found that the mutations in these strains reduce the translational efficiency of the T7 RNA polymerase [59]. C41 and C43 are currently commercially available by Avidis.

Recently, Bowie and co-workers used a combination of the mutagenic base analog 2-aminopurine and the mutator gene *mutD5 *(a mutated *dnaQ *gene causing a DNA proofreading defect), to evolve *E. coli *strains which accumulate markedly enhanced amounts of a variety of different *Mycobacterium tuberculosis *rhomboid family proteins and other prokaryotic and eukaryotic integral membrane proteins [60]. These strains were found to produce up to 90-fold higher amounts of protein compared to the parental strain TOP10. In an analogous manner, our group has used the chemical mutagen *N*-methyl-*N'*-nitro-*N*-nitrosoguanidine to generate *E. coli *mutants that confer up to 5-fold greater yields of properly assembled full-length IgG antibodies in the bacterial periplasm [61]. In another example of classical strain mutagenesis for enhanced recombinant protein production, Skretas and Georgiou used insertional mutagenesis of the Tn*5 *transposon together with fluorescence-activated cell sorting (FACS), to isolate *E. coli *MC4100A variants that accumulate increased amounts of the membrane-inserted human GPCR central cannabinoid receptor (CB1) [62].

Genes, gene fragments or operon fragments that favorably affect protein expression can be isolated from plasmid libraries co-expressing genomic fragments. Alternatively, individual intact genes can be identified using the ASKA library, an ordered library of all the *E. coli *ORFs transcribed from the inducible T5*lac *promoter [63]. Using this library, our group identified *E. coli *proteins that enhanced the yields of the membrane-embedded form of the human GPCR bradykinin receptor 2 (BR2) [64]. One of these, the putative DNA-binding protein of unknown function YbaB, conferred a ~10-fold increase in the accumulation of membrane-integrated and folded BR2, as well as a variety of membrane proteins tested of either prokaryotic or eukaryotic origin.

The described genetic engineering strategies for enhancing recombinant protein production in bacteria are summarized in Table 1.

**Table 1:** Genetic engineering strategies which have been applied to the enhancement of recombinant protein production in bacteria

Method	Strain	Target protein	Reference
Spontaneous chromosomal mutagenesis	C(1(DE3), C43(DE3)	Bovine OGCP; bovine phosphate carrier; bovine ADP/ATP translocase; *Bacillus subtilis *PS3 alanine/H^+ ^carrier; *E. coli *F-ATPase subunits b and c; bovine F-ATPase subunits b, d, α, β, γ, δ, OSCP, F_6_; bovine F-ATPase inhibitor protein; *Aequoria victoria *GFP	[58]

Chromosomal mutagenesis using chemical mutagens or mutator genes	EXP-Rv1337-1, EXP-Rv1337-2, EXP-Rv1337-3, EXP-Rv1337-4, EXP-Rv1337-5	*Mycobacterium tuberculosis *Rv1337, *M. tuberculosis *Rv2746, *M. tuberculosis *Rv2835, *M. tuberculosis *Rv0110, *Methanocccus jannaschii *rhomboid (MJR), *Drosophila melanogaster *rhomboid 1 (Rho1)	[60]
	
	GS1, TM1, TM2, TM3, TM4, TM5, TM6	Variants of human IgG1	[61]

Transposon mutagenesis	GS101 (MC4100A *dnaJ*349::Tn*5 *(Kan^R^)) GS102 (MC4100A *dinG*1377::Tn*5 *(Kan^R^)) GS103 (MC4100A *nhaR*63::Tn*5 *(Kan^R^)) GS104 (MC4100A Δ*dinG*) GS105 (MC4100A Δ*dinG dnaJ*349::Tn*5 *(Kan^R^))	Human central cannabinoid receptor (CB1)	[62]

Co-expression of the ASKA library	MC4100A (+*ybaB*) MC4100A (+*yciQ*) MC4100A (+*glpQ*)*	Human bradykinin receptor 2 (BR2), CB1, human neurokinin (substance P) receptor 1 (NKR1), *E. coli *YidC, *E. coli *CstA, human stearoyl-CoA desaturase (SCD)	[64]

*MC 100A (+*name of gene*) denotes an MC 100 strain overexpressing the *E. coli *gene specified in parentheses.

#### 2.2 Genome engineering

Genome engineering techniques refer loosely to a group of methods for introducing desired genetic diversity within known regions of the chromosome. Modifying the transcriptional landscape of *E. coli*, e.g. by generating libraries of randomized transcription factors or by mutating components of the RNA polymerase, is an effective means of generating complex phenotypes. Although genome engineering has not yet been applied extensively to the optimization of recombinant protein expression, it holds great promise for the creation of the next generation of *E. coli *host strains for protein production. The great advantage of these methods is that they can have a global impact on cellular pathways and physiology [65]. Examples of genome engineering methods likely to be of particular interest for expression optimization are outlined below and summarized in Table 2.

**Table 2:** Representative genome engineering strategies which could be applied to the enhancement of recombinant protein production in bacteria

Method	Targeted cellular component	Target organism	Engineered phenotype	References
Global transcription machinery engineering (gTME)	General sigma factor σ^70^, stationary phase sigma factor σ^S^, RNA polymerase a subunit	*E. coli*	Ethanol, butanol, isobutanol, pentanol, and 3-pentanol tolerance; lycopene, L-tyrosine, and hyaluronic acid production	[55,66,68]
	
	Transcription factor Spt15p	*S. cerevisiae*	Ethanol tolerance and production	[67]
	
	General sigma factor	*Lactobacillus plantarum*	lactic acid and hydrochloric acid tolerance	[95]

Libraries of artificial zinc fingers	Zinc fingers	*S. cerevisiae*	Tolerance to heat and osmotic stress; ketoconazole resistance	[69]
		
		Mouse neuroblastoma cells	Neurogenesis, differentiation of neuroblasts to osteoblasts, proliferation rate	[69]
		
		*E. coli*	Tolerance to heat, cold, and osmotic stress	[70,71]
		

Trackable multiplex recombineering (TRMR)	> 95% of all individual *E. coli *genes	*E. coli*	Tolerance to salicin, D-fucose, methylglyoxal, valine, and lignocellulosic hydrolysate	[56]

Genome shuffling	Chromosome	*Streptomyces fradiae*	Tylosin production	[72]
		
		A strain of *Lactobacillus*	Tolerance to lactic acid	[73]
		
		*Sphingobium chlorophenolicum*	Degradation of pentachlorophenol	[74]

Global transcription machinery engineering (gTME) is a new tool that enables the reprogramming of the cellular transcriptome through random mutagenesis (e.g. by error-prone PCR) of selected components of the transcriptional machinery, such as the *E. coli *sigma factor σ^70^, the α subunit of the *E. coli *RNA polymerase, or the *S. cerevisiae *TATA-binding transcription factor Spt15p. Screening of plasmid-encoded gTME libraries was used to isolate strains with increased tolerance to alcohols and for enhanced production of small molecules, such as lycopene (50% increase), L-tyrosine (150% increase), hyaluronic acid (60% increase), and others [55,66-68].

Zinc fingers are highly specific DNA-binding protein domains that recognize three-base-pair sequences and are found in a variety of transcriptional regulatory proteins. A single transcription factor can include several of these motifs, which can be assembled in a highly modular fashion to target loner motifs and confer sequence selectivity. Fusions of random combinations of zinc fingers with activator or repressor domains have been employed to introduce high levels of diversification of transcription, which in turn can generate diverse complex phenotypes, such as tolerance to high and low temperatures, drug resistance, osmotic tolerance, and differentiation in different organisms, such as *Saccharomyces cerevisiae*, mammalian cells, and *E. coli *[69-71]. Such libraries of random combinations of zinc fingers can potentially be used to generate engineered bacterial strains, whose evolved transcriptome affects favorably recombinant protein production.

Very recently, Gill and co-workers have developed a creative methodology, termed trackable multiplex recombineering (TRMR), for constructing libraries of genetically modified microorganisms based on homologous recombination of pools of synthetic oligos [56]. Briefly, two sets of oligoDNA cassettes were synthesized: Each contained 5' and 3' recognition sequences for homologous recombination of the ribosome-binding site (RBS) of each one of the 4,077 protein-coding genes of *E. coli *MG1655, interrupted by a gene-specific tracking sequence, an antibiotic resistance marker for selection of successful recombination, and an "up cassette" or a "down cassette". The "up cassette" contained the sequences of the strong inducible promoter P_LtetO-1 _with a RBS, whose function was to generally up-regulate the expression of its target gene, while the "down cassette" contained an inert sequence, whose function was to generally down-regulate gene expression. Homologous recombination of these oligonucleotides enabled the creation of pools of bacteria displaying upregulation or downregulation of genes at a genomic scale. The library of the mutant strains (2 × 4,077 = 8,154 total) was subsequently subjected to selection for growth under various conditions. Using this approach, Warner et al. reported the isolation of thousands of clones with improved growth phenotype in various conditions within a week [56]. The isolated clones could be easily characterized by sequencing or by microarray analysis using the recombined tag sequences to identify the genes responsible for the evolution of these complex phenotypes. However, it is not clear yet if the TRMR libraries of bacterial cells with up- and down-regulated genes will enable the evolution of novel traits which are different than those achieved with the use of the ASKA library and the Keio collection, respectively.

Once a collection of strains displaying increased expression has been created by one of the techniques discussed so far, whole genome recombination or "shuffling" may be employed to create a library of clones containing combinations of alleles that contribute to better expression. Strains containing combination of alleles that act synergistically can then be isolated [72]. Consecutive rounds of genome shuffling have been shown to result in the rapid emergence of complex phenotypes in a variety of microorganisms, such as a nine-fold improvement of tylosin production in *Streptomyces fradiae*, a three-fold increase in lactic acid production in a poorly characterized industrial strain of *Lactobacillus*, and a dramatically enhanced ability to degrade the anthropogenic pesticide pentachlorophenol in *Sphingobium chlorophenolicum *[72-74]. Genome shuffling in *E. coli*, however, is rather inefficient [75] and, therefore, new techniques will have to be developed before this methodology becomes routine for this organism.

#### 2.3 Screening/Selection platform

An important issue in the engineering of novel strains for improved expression is how to monitor the yield of the desired protein in a high-throughput manner. For small libraries, microtiter well plates can be used to screen up to a few thousand clones. Immunoassays, namely enzyme-linked immunosorbent assays (ELISAs) and 96-well Western blot analyses can be used to quantify the level of soluble protein when no functional assay is available. However, screening of libraries sizes exceeding ~10^5 ^clones requires the use of single-cell assay formats [76]. Designing the appropriate selection or screening process for the isolation of clones with the desired phenotypes is a key factor for the implementation of genome engineering strategies for enhanced recombinant protein production. A number of high-throughput selection/screening systems have been developed and/or utilized in the past few years for the development of such overexpressing strains.

##### 2.3.1 Genetic selection

The levels of accumulation of a protein of interest can be coupled with the growth of the host cell under selective conditions by expressing the target protein in the form of a chimeric fusion with a reporter protein which exhibits a selectable phenotype, such as an antibiotic resistance marker. Bowie and coworkers, for example, isolated *E. coli *strains with enhanced capacity for integral membrane protein expression by selecting for antibiotic resistance conferred by expressing two separate C-terminal fusions of the *M. tuberculosis *rhomboid membrane protein Rv1337 to chroramphenicol acetyltransferase (the enzyme conferring resistance to the antibiotic chloramphenicol) or aminoglycoside 3'-phosphotransferase (the enzyme conferring resistance to the antibiotic kanamycin) [60]. Our group has developed a simple genetic selection system for enhanced recombinant membrane protein production in *E. coli*, by utilizing a tripartite fusion comprising the human GPCR BR2 with an N-terminal DsbA leader sequence, which targets the recombinant protein to the signal recognition particle pathway for insertion into the bacterial inner membrane, and a C-terminal β-lactamase [64]. A number of similar approaches have been developed using chloramphenicol acetyltransferase [77,78] and dihydrofolate reductase (DHFR) [79], or combinations of these [80] as fusion reporter proteins.

Recently, protein fragment complementation assays were developed especially for monitoring protein folding and expression. In this systems, the protein of interest is inserted into the middle of a reporter gene, such as β-gatactosidase [81], β-lactamase [82], or GFP [83-85]. Since the activity of the reporter is designed to be recovered only when the correct folding of the test protein has occurred, its activity is proportional to the level of accumulation of correctly folded protein in the cell.

Recently, DeLisa and colleagues developed a novel selection platform for protein folding, by capitalizing on the properties of the bacterial twin-arginine translocation (Tat) pathway [86]. The bacterial Tat pathway is a Sec-independent inner membrane transport system that is known for its ability to transport only proteins that have undergone folding before translocation [87]. In this system, a protein of interest is inserted between an N-terminal Tat signal peptide and a C-terminal β-lactamase enzyme. Since β-lactamase is active when it is exported into the periplasm, only cells with correctly folded target protein can survive on antibiotic-containing selective media.

##### 2.3.2. High-throughput screening using fluorescent reporters

Since the original observation by Waldo and co-workers that the fluorescence of *E. coli *cells expressing a C-terminal fusion of a recombinant protein with the green fluorescent protein (GFP) correlates well with the expression levels of well folded and soluble protein [88], fluorescent proteins have been widely used to monitor the expression level for both soluble and membrane-embedded proteins [7,62,89,90]. Microplates using a fluorescence plate reader, dot blot analyses using a fluorescence scanner, or flow cytometry are routinely used for monitoring the fluorescence of GFP fusions [91-93]. Flow cytometry is by far the most powerful tool for fluorescence-based library screening in terms of throughput, ability to monitor fluorescence at the single-cell level in a quantitative manner, and the isolation of desired clones [7,62,76,89].

**Figure 1:**
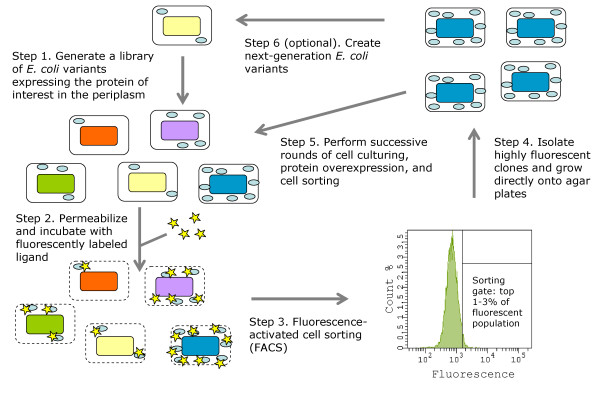
**Periplasmic expression with cytometric sorting (PECS) for enhanced recombinant protein expression**. *E. coli *cells expressing the protein of interest in the periplasm are incubated in a high-osmolarity buffer that renders their outer membrane permeable to a fluorescently labeled ligand. Cell fluorescence is proportional to the number of functional, ligand-binding molecules in the periplasm. Clones containing genetic lesions that increase protein expression, display higher fluorescence and can be rapidly isolated using FACS. Adapted from Makino et al. [61].

The accumulation of active, secreted protein at the single-cell level can be readily monitored by periplasmic expression followed by cytometric sorting (PECS) [94]. In this technique, *E. coli *cells expressing a protein in the periplasm are incubated in a high-osmolarity buffer that renders their outer membrane permeable to a ligand labeled with a fluorescent probe (Figure 1) [94]. The fluorescent ligand binds to the properly folded protein, conferring cell fluorescence proportional to the amount of functional protein in the periplasm. Clones containing mutations that increase the expression of functional protein, display higher fluorescence and can be isolated by FACS. By using this technique, we have isolated several *E. coli *mutant strains which accumulate markedly enhanced quantities of full-length and properly assembled IgG antibodies in the bacterial periplasm [61]. Furthermore, we have utilized PECS to isolate several genes and gene clusters which confer high expression levels of properly folded integral membrane proteins, including several mammalian GPCRs and native bacterial membrane proteins [GS, TM, Navin Varadarajan, Mark Pogson, and GG; manuscript in preparation].

## Conclusion

Recent studies have demonstrated that strain/genetic engineering is a very promising approach for evolving engineered *E. coli *strains with markedly enhanced capacities for recombinant protein production. Several unique and powerful methods have emerged recently that allow the generation of large libraries of bacterial mutants carrying different types of genetic profiles. Furthermore, advances in high-throughput screening have enabled the monitoring of the overexpression phenotype at the single-cell level and the rapid isolation of the rare clones with the desired overexpression profiles. The information obtained from the analysis of the genetic profiles in the isolated strains can provide invaluable and fundamental understanding about the biology of protein biogenesis, folding, stability and homeostasis in bacteria. These pieces of information can subsequently be combined and utilized to generate specialized protein expression bacterial "cell factories" for uses in research as well as in the industrial field.

## Competing interests

The authors declare that they have no competing interests.

## Authors' contributions

All authors defined the topic of the review and wrote, read and approved the manuscript.
